# Supplementary material for: Proteomics Readjustment of the Yarrowia lipolytica Yeast in Response to Increased Temperature and Alkaline Stress
Source: Microorganisms. 2021 Dec 18;9(12):2619. doi: 10.3390/microorganisms9122619 (PMC8708323; doi:10.3390/microorganisms9122619)
Supplement: Supplementary file 1 [file microorganisms-09-02619-s001.zip › Figures S1-S4.pdf]

Figure S1 QR-code of the availability to the complete list of the proteins.

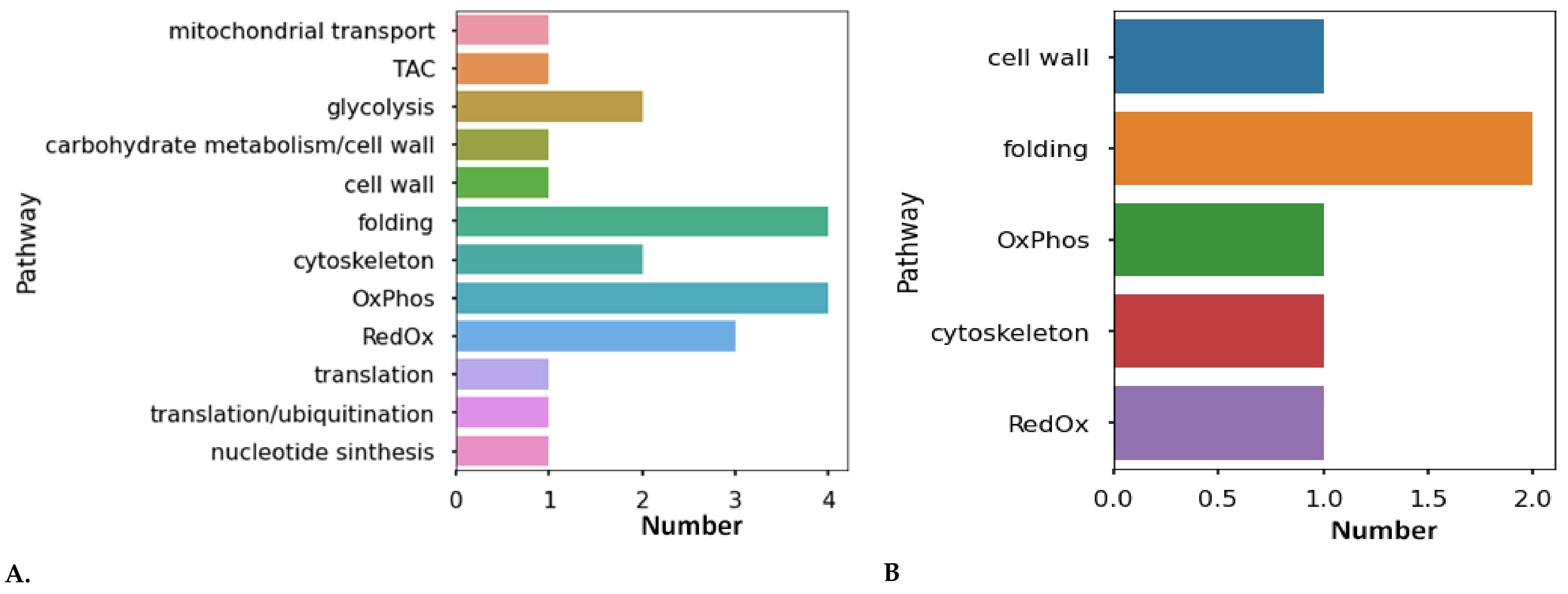

Figure S2. The number of the identified proteins (x-axis) involved in different metabolic pathways under the normal conditions: A- total proteins; B – unique proteins. The identified differentially expressed proteins were divided into different cellular processes using the Blast2GO pro software (http://www.blast2go.com/; accessed on October, 26, 2021, version 3.2). Thirteen cellular processes were identified in the normal conditions.

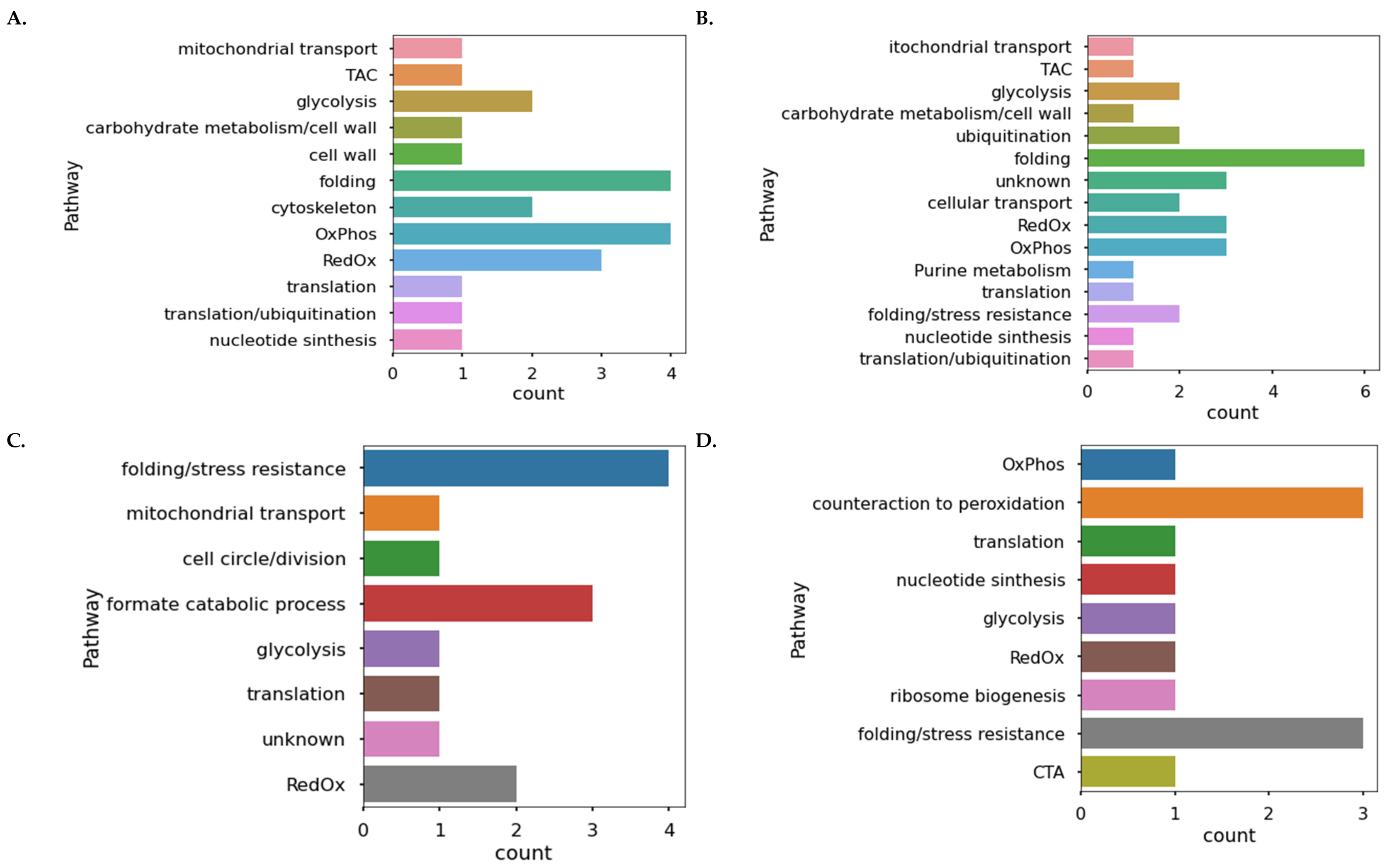

Figure S3. The number of the identified proteins (x-axis) involved in different metabolic pathways at the alkaline stress: A- total proteins; B – unique proteins; C – upregulated proteins; D – down-regulated proteins. Distribution of differentially expressed proteins due to various biological processes. The identified differentially expressed proteins were divided into different cellular processes using the Blast2GO pro software (http://www.blast2go.com/, accessed on October, 26, 2021, version 3.2). Sixteen cellular processes were identified upon the adaptive response to ambient alkaline challenge.

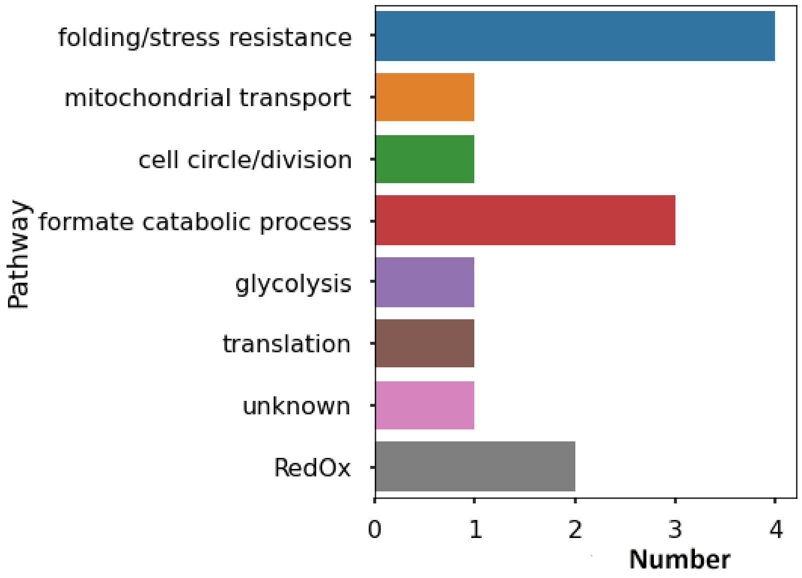

A

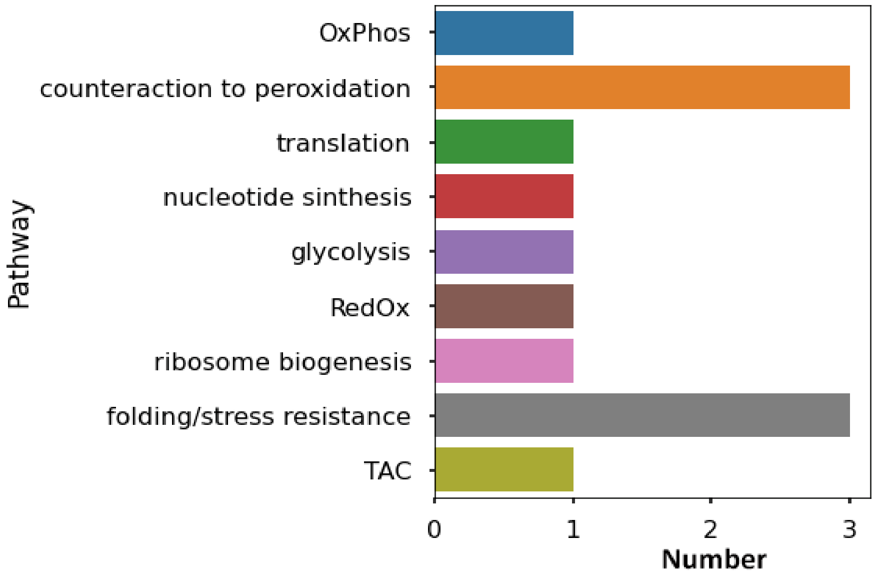

B

Figure S4. The number of the identified original proteins (x-axis) involved in different metabolic pathways at the heat (A) and combined (B) types of stress. The distribution of identified differentially expressed proteins were divided into different cellular processes using the Blast2GO pro software (http://www.blast2go.com/, accessed on October, 26, 2021, version 3.2). Eight cellular processes were identified upon the adaptation to the heat shock and nine ones were in the response to the combined stress.
