# Supplementary material for: Proteomics Readjustment of the Yarrowia lipolytica Yeast in Response to Increased Temperature and Alkaline Stress
Source: Microorganisms. 2021 Dec 18;9(12):2619. doi: 10.3390/microorganisms9122619 (PMC8708323; doi:10.3390/microorganisms9122619)
Supplement: Supplementary file 1 [file microorganisms-09-02619-s001.zip › Table S2.pdf]

Table S2 (linked to Figure 4)

A: The list of the proteins interacting with the up-regulated HSP60 heat shock protein (YALIOF02805p)

YALI0B05610p	*Uncharacterized protein; YALI0B05610p; Belongs to the GroES chaperonin family*	0.999
YALI0C17347p	*Uncharacterized protein; YALI0C17347p; Belongs to the heat shock protein 70 family*	0.996
YALI0C07953p	*Uncharacterized protein; YALI0C07953p*	0.982
KAR2	*Uncharacterized protein; YALI0E13706p; Belongs to the heat shock protein 70 family*	0.964
YALI0C18513p	*Essential component of the PAM complex, a complex required for the translocation of transit peptide-containing proteins from the inner membrane into the mitochondrial matrix in an ATP-dependent manner*	0.935
ATP2	*Mitochondrial membrane ATP synthase (F(1)F(0) ATP synthase or Complex V) produces ATP from ADP in the presence of a proton gradient across the membrane which is generated by electron transport complexes of the respiratory chain . F-type ATP synthases consist of two structural domains, F(1) - containing the extramembraneous catalytic core, and F(0) - containing the membrane proton channel, linked together by a central stalk and a peripheral stalk . During catalysis, ATP synthesis in the catalytic domain of F(1) is coupled via a rotary mechanism of the central stalk subunits to proton tr [...]*	0.933
YALI0E13255p	*Uncharacterized protein; YALI0E13255p; Belongs to the heat shock protein 70 family*	0.923
YALI0F31207p	*Uncharacterized protein; YALI0F31207p*	0.915
YALI0E21307p	*Belongs to the cytochrome b5 family.*	0.913
YALI0E15268p	*Uncharacterized protein; YALI0E15268p*	0.905

B: The list of the proteins interacting with the up-regulated Peptidyl-prolyl cis-trans isomerase (YALIOC10230p)

Q6C3C8	*YALI0F00770p*	0.917
Q6CDY4	*YALI0B20262p*	0.917
Q6CCW1	*Histone deacetylase; Belongs to the histone deacetylase family. HD Type 1 subfamily.*	0.913
Q6C330	*YALI0F03069p*	0.912
Q6C219	*YALI0F11583p*	0.814
FPR1	*FK506-binding protein 1; PPIases accelerate the folding of proteins. It catalyzes the cis-trans isomerization of proline imidic peptide bonds in oligopeptides (By similarity); Belongs to the FKBP-type PPIase family. FKBP1 subfamily.*	0.813
Q6C237	*YALI0F11165p*	0.795
Q6C2D7	*YALI0F08745p*	0.784
Q6C1D2	*YALI0F17314p*	0.762
Q6C7S4	*YALI0D25762p*	

C: The list of the proteins interacting with the up-regulated Glyceraldehyde-3-phospate dehydrogenase (YALIOC06369p)

	Belongs to the triosephosphate isomerase family.	0.999
YALI0E26004p	*Catalyzes the aldol condensation of dihydroxyacetone phosphate (DHAP or glycerone-phosphate) with glyceraldehyde 3-phosphate (G3P) to form fructose 1,6-bisphosphate (FBP) in gluconeogenesis and the reverse reaction in glycolysis*	0.999
PGK1	*Phosphoglycerate kinase; YALI0D12400p; Belongs to the phosphoglycerate kinase family*	0.999
ENO	*Uncharacterized protein; YALI0F16819p*	0.993
YALI0F07711p	*Glucose-6-phosphate isomerase; YALI0F07711p; Belongs to the GPI family*	0.991
YALI0F15587p	*Transaldolase is important for the balance of metabolites in the pentose-phosphate pathway*	0.983
YALI0E22649p	*Catalyzes the rate-limiting step of the oxidative pentose- phosphate pathway, which represents a route for the dissimilation of carbohydrates besides glycolysis*	0.979
YALI0F01584p	*Triosephosphate isomerase; YALI0F01584p*	0.976
YALI0E06479p	*Catalyzes the transfer of a two-carbon ketol group from a ketose donor to an aldose acceptor, via a covalent intermediate with the cofactor thiamine pyrophosphate*	0.969
YALI0D02277p	*Uncharacterized protein; YALI0D02277p*	0.964

D: The list of the proteins interacting with the up-regulated Triosephospate isomerase(YALIOF05214p)

Q6C2I1	*Glucose-6-phosphate isomerase; Belongs to the GPI family.*	0.999
Q6C1K2	*Transaldolase; Transaldolase is important for the balance of metabolites in the pentose-phosphate pathway.*	0.998
GPD	*Glyceraldehyde-3-phosphate dehydrogenase; Belongs to the glyceraldehyde-3-phosphate dehydrogenase family.*	0.998
Q6CFX7	*Phosphoglycerate mutase; Belongs to the phosphoglycerate mutase family. BPG- dependent PGAM subfamily.*	0.997
Q6C4K5	*Fructose-bisphosphate aldolase; Catalyzes the aldol condensation of dihydroxyacetone phosphate (DHAP or glycerone-phosphate) with glyceraldehyde 3-phosphate (G3P) to form fructose 1,6-bisphosphate (FBP) in gluconeogenesis and the reverse reaction in glycolysis; Belongs to the class II fructose-bisphosphate aldolase family.*	0.996
Q6C395	*Triosephosphate isomerase*	0.993
ENO	*Enolase*	0.990
PGK1	*Phosphoglycerate kinase; Belongs to the phosphoglycerate kinase family.*	0.988
Q6C6T4	*Transketolase; Catalyzes the transfer of a two-carbon ketol group from a ketose donor to an aldose acceptor, via a covalent intermediate with the cofactor thiamine pyrophosphate.*	0.988
Q6C394	*YALI0F01606p*	0.976

E: The list of the up-regulated proteins interacting with VDAC (YAL10F17314p)

YALI0F31207p	*Uncharacterized protein; YALI0F31207p*	0.964
YALI0B10362p	*Uncharacterized protein; YALI0B10362p*	0.954
YALI0A07084p	*Uncharacterized protein; YALI0A07084p*	0.954
GEM1	*Mitochondrial GTPase involved in mitochondrial trafficking. Probably involved in control of anterograde transport of mitochondria and their subcellular distribution*	0.945
QCR2	*Uncharacterized protein; YALI0F08613p; Belongs to the peptidase M16 family*	0.910
YALI0F08899p	*Uncharacterized protein; YALI0F08899p; Belongs to the TRAFAC class dynamin-like GTPase superfamily. Dynamin/Fzo/YdjA family*	0.893
ATP1	*Mitochondrial membrane ATP synthase (F(1)F(0) ATP synthase or Complex V) produces ATP from ADP in the presence of a proton gradient across the membrane which is generated by electron transport complexes of the respiratory chain . F-type ATP synthases consist of two structural domains, F(1) - containing the extramembraneous catalytic core, and F(0) - containing the membrane proton channel, linked together by a central stalk and a peripheral stalk . During catalysis, ATP synthesis in the catalytic domain of F(1) is coupled via a rotary mechanism of the central stalk subunits to proton tr [...]*	0.892
YALI0C11011p	*Uncharacterized protein; YALI0C11011p*	0.888
MDM10	*Component of the ERMES/MDM complex, which serves as a molecular tether to connect the endoplasmic reticulum and mitochondria. Components of this complex are involved in the control of mitochondrial shape and protein biogenesis and may function in phospholipid exchange. MDM10 is involved in the late assembly steps of the general translocase of the mitochondrial outer membrane (TOM complex). Functions in the TOM40-specific route of the assembly of outer membrane beta-barrel proteins, including the association of TOM40 with the receptor TOM22 and small TOM proteins. Can associate with the [...]*	0.864
YALI0F03567p	*Uncharacterized protein; YALI0F03567p*	0.835

F: The list of the up-regulated proteins interacting with malate dehydrogenase (YAL10D16753p

YALI0E02684p	*Citrate synthase; YALI0E02684p; Belongs to the citrate synthase family*	0.999
YALI0C06776p	*Uncharacterized protein; YALI0C06776p*	0.999
YALI0B02178p	*Aspartate aminotransferase; YALI0B02178p*	0.998
YALI0F29337p	*Aspartate aminotransferase; YALI0F29337p*	0.996
YALI0E00638p	*Citrate synthase; YALI0E00638p; Belongs to the citrate synthase family*	0.996
YALI0E18634p	*Uncharacterized protein; YALI0E18634p*	0.987
YALI0C16995p	*Uncharacterized protein; YALI0C16995p*	0.987
YALI0C24101p	*Catalyzes a 2-step reaction, involving the ATP-dependent carboxylation of the covalently attached biotin in the first step and the transfer of the carboxyl group to pyruvate in the second.*	0.980
YALI0E15708p	*Malate synthase; YALI0E15708p; Belongs to the malate synthase family*	0.977
YALI0D19140p	*Malate synthase; YALI0D19140p; Belongs to the malate synthase family*	0.977
