# Supplementary material for: Proteomics Readjustment of the Yarrowia lipolytica Yeast in Response to Increased Temperature and Alkaline Stress
Source: Microorganisms. 2021 Dec 18;9(12):2619. doi: 10.3390/microorganisms9122619 (PMC8708323; doi:10.3390/microorganisms9122619)
Supplement: Supplementary file 1 [file microorganisms-09-02619-s001.zip › Table S3.pdf]

Table S3 (linked to Figure 5)

A: The list of the induced proteins interacting with SSA4 heat shock protein of HSP70 family

Q6C3C3	*YALI0F00880p*	0.989
Q6C618	*YALI0E13255p*	0.988
Q6CCI8	*YALI0C08987p*	0.983
Q6CCN4	*YALI0C07953p*	0.975
Q6CI89	*YALI0A00594p*	0.970
Q6CEW3	*YALI0B12408p*	0.969
Q6CGR2	*Clathrin heavy chain; Clathrin is the major protein of the polyhedral coat of coated pits and vesicles; Belongs to the clathrin heavy chain family.*	0.964
Q6C608	*YALI0E13508p*	0.958
Q6CAS1	*YALI0D00429p*	0.958
Q6C4W1	*YALI0E23265p*	0.949

B: The list of the induced proteins interacting with Thioredoxin (CPAR2_200490)

G8B9M8	*Thioredoxin reductase*	0.999
G8BJ65	*Thioredoxin domain-containing protein*	0.999
G8BEE7	*PMSR domain-containing protein*	0.990
G8BHL6	*Glutathione reductase; Maintains high levels of reduced glutathione in the cytosol. ; Belongs to the class-I pyridine nucleotide-disulfide oxidoreductase family.*	0.986
G8BE75	*Uncharacterized protein*	0.983
G8BDM1	*Sulfiredoxin*	0.972
G8BH83	*Superoxide dismutase [Cu-Zn]; Destroys radicals which are normally produced within the cells and which are toxic to biological systems. ; Belongs to the Cu-Zn superoxide dismutase family.*	0.964
G8BH89	*Superoxide dismutase [Cu-Zn]; Destroys radicals which are normally produced within the cells and which are toxic to biological systems. ; Belongs to the Cu-Zn superoxide dismutase family.*	0.964
G8B642	*Peptide-methionine (R)-S-oxide reductase; Belongs to the MsrB Met sulfoxide reductase family.*	0.959
G8BJ84	*BZIP domain-containing protein*	0.951
